# Gender Effects in Young Road Users on Road Safety Attitudes, Behaviors and Risk Perception

**DOI:** 10.3389/fpsyg.2016.01412

**Published:** 2016-09-27

**Authors:** Pierluigi Cordellieri, Francesca Baralla, Fabio Ferlazzo, Roberto Sgalla, Laura Piccardi, Anna Maria Giannini

**Affiliations:** ^1^Department of Psychology, “Sapienza” University of RomeItaly, Rome; ^2^Department of Public Security, Ministry of InteriorItaly, Rome; ^3^Department of Life, Health and Environmental Sciences, L'Aquila UniversityL'Aquila, Italy, Rome; ^4^Neuropsychology Unit, IRCCS Santa Lucia FoundationItaly, Rome

**Keywords:** risk perception, worry, driving behavior, sex differences, young drivers

## Abstract

In the present study, we investigated gender-related effects on road safety attitudes in 2681 young drivers (1458 males, 54.4%; aged 18–22) who filled out several scales assessing attitudes toward road safety issues, driving behavior in specific hypothetical situations, accident risk perception, and concerns about such a risk. We focused only on young drivers to better understand the role of gender in road safety attitudes in a period of life in which risky behaviors are widespread for males and females. Indeed, there is still no agreement as to the nature of these gender differences. According to some authors, the effects of gender on being involved in a crash due to driving skills are either non-existent or largely explained by differences in alcohol consumption. In our study, we found gender differences in road safety attitudes (i.e., “negative attitude toward traffic rules and risky driving”; “negative attitude toward drugs and alcohol” and “tolerance toward speeding”) and in driver behavior (i.e., “errors in inattentive driving” and “driving violations”). This result is consistent in all drivers coming from nine different European countries. Our analyses yielded an important finding concerning risk perception. The results indicate that the level of risk perception during driving is the same for males and females. However, these two groups differ in the level of concern about this risk, with males being less concerned about the risk of a road accident. This suggests that the main difference between these two groups is not strictly related to judgment of the perceived risk probability but rather to the level of concern experienced about the consequences of the risk. This difference between risk perception and worry could explain differences in the frequency of car accidents in the two groups. The present findings may provide new insights for the development of gender-based prevention programs.

## Introduction

Every year, many people worldwide are killed or severely injured in motor vehicle accidents (WHO, 2015; OECD, 2016). On the front line, road authorities all over Europe are trying different alternatives to change driver behavior to reduce road accidents and general costs to society. To investigate this phenomenon in depth, including a better understanding of the relationship between demographic factors (i.e., gender, educational level, age), personal attitudes and beliefs concerning driving behavior and dangerous driving, several contributions have evaluated the importance of “human factors” (Grayson and Maycock, 1988; Lajunen, 1997) in driving behavior (Lourens et al., 1999). The evaluation of driving behavior and driver's skills and their role in car accidents is particularly complex. The focus of behavioral factors in road safety research was initially approached by evaluating driving abilities and expertise in relation to the age of drivers (i.e., Matthews and Moran, 1986). Subsequently, research focused on willingness to take risks (i.e., risky driving behavior and the role of sensation seeking: Zuckerman and Neeb, 1980; Jonah, 1997; the determinants of risky driving behavior: Parker et al., 1992, 1998; Rutter et al., 1995), underestimating the risk while driving (Taubman-Ben Ari et al., 2004; Delhomme et al., 2009) and overestimating their driving skills (Kruegar and Dickson, 1994; Horswill et al., 2004).

Gender has been considered in relation to risky driving behavior in young drivers (Ulleberg and Rundmo, 2003; Teese and Bradley, 2008) and in general, it has been found that, in terms of risk behavior in road traffic, males are more willing to take risks than female (Whissell and Bigelow, 2003; Oltedal and Rundmo, 2006). Yagil (1998) has reported that the rate of men's involvement in fatal road accidents is twice as high as women's and, previously, Evans (1991) reported that a woman has a 25% less chance than a man to be involved in a road accident. Furthermore, according to other authors, men are involved in road accidents as a consequence of their violation of traffic laws (i.e., violations of speed limits and driving after drinking: Storie, 1977; Simon and Corbett, 1996; Harre et al., 1996), whereas women were involved in road accidents due to judgment errors (Storie, 1977). It has been furthermore found that women take fewer risks than men do when driving (Ebbesen and Haney, 1973; Katz et al., 1975).

Among demographic factors, age is another negative predictor of risky driving behavior. It has been well established by studies and accident databases from various countries that young novice drivers are more frequently involved in traffic accidents than drivers in other age groups (WHO, 2015; OECD, 2016). In general, a variety of factors, such as inadequate skills and/or a greater propensity to assume more risk, have frequently been indicated as the main causes of accidents in this age group (Deery, 1999; Underwood, 2007; Giannini et al., 2013). Although it is unanimously recognized that young people are more at risk than other age groups, it is unclear whether there are gender differences within this age group. Some studies found that young male drivers are more involved in road accidents (Arnett, 2002), aggressive driving (Simon and Corbett, 1996), and violation of traffic and road laws (Jonah and Dawson, 1987; Fletcher, 1995). Analysing risky attitudes, Matthews and Moran (1986) found that young male drivers tend to see themselves as relatively immune to the hazards threatening their peers. Moreover, Glendon et al. (1996) found that young male drivers tend to underestimate their own personal risk perception and overestimate their competence when compared to females.

However, more recent studies report that female drivers are now over-represented in crashes compared to males, due to errors in yielding, gap acceptance, and speed regulations (Classen et al., 2012). Laapotti et al. (2001, 2003) found that although females have a greater safety orientation than males, young female drivers show more problems in vehicle handling and mastering traffic situations.

Taking this evidence altogether suggests that age is a crucial demographic factor in terms of presence/absence of gender differences (Rhodes and Pivik, 2011). Recently, Lucidi et al. (2010) identified different types of young drivers' profiles in a large sample of 1800 young men and women 18–23 years of age with a valid driver's license. They classified three different profiles in detail (i.e., risky drivers; worried drivers and careful drivers) and found that the 75.4% of “risky drivers” are male.

Kelley-Baker and Romano (2010) reported that in the United States, the prevalence of women involved in fatal motor vehicle accidents is rising, while it is decreasing among men. Romano et al. (2008) observed that this increase could be mostly due to an increase in traffic exposure, as well as to an increase of riskier driving behavior in women, particularly in young female drivers. Furthermore, Kelley-Baker and Romano (2010) found that many of the gender-based differences associated to skill-related crashes were either non-existent or largely explained by gender differences in alcohol consumption. Therefore, the role of gender as a predictive factor in risky driving behavior deserves further investigation. Generally speaking, socio-cultural dynamics produce different opportunities to learning in males and in females. In the past, one negative consequence of these dynamics was to be encompassed in the so-called stereotype threat in which people are or feel themselves to be at risk of confirming negative stereotype about their group (Inzlicht and Schmader, 2012). Educational system, messages spread by mass media and society in general contribute to the diffusion of implicit information regarding sex roles in young people (Rolandelli, 1991) explaining also why young drivers have high risky behaviors of older drivers. It is also possible that scientific researches that have reported that male drivers are more prone to suffer from crashes have produced changes in women and men's self-belief and perceptions about driving behaviors interlaced with general society changes. This is in line with the above mentioned findings in which the prevalence of women involved in fatal motor-vehicle crashes is rising (e.g., Kelley-Baker and Romano, 2010). In the present study, we investigated gender-related effects on road safety attitudes.

As several studies have found an interactive effect of gender and age on driving behavior, our sample included only young drivers aged 18–22 years. We focused on young drivers because, as demonstrated by Gregersen and Bjurulf (1996), Maycock et al. (1991), Brown and Groeger (1988), and Deery (1999), they are more likely to underestimate the risk of being involved in a crash and to overestimate their own abilities as drivers. A possible explanation of this tendency could be found in a general tendency toward risky behavior regardless of the driving situation (e.g., Jessor, 1987). For such a reason, we investigated both the attitude toward risk and the risk perception for better understanding whether the presence of risky behaviors could be related to a deficit in the actual risk perception.

To our knowledge this is the first time that the investigation have been performed enrolling young drivers from nine different European countries, generally the topic is analyzed in just one country at a time. This large sample may allow understanding the role of gender in road safety attitudes in young drivers in a large geographical area.

## Methods

### Participants

A preliminary sample of 2681 young individuals from Italy, Austria, Bulgaria, Cyprus, Germany, Ireland, Latvia, Lithuania, and Poland participated in the study (1458 males, 54.4%, age range 18–22 years; Table 1). Participants were recruited from schools and universities that had been preliminarily selected at random from each different country according to different demographic districts. School classes and university courses were then randomly selected from each institute and students agreed or disagreed to participate.

**Table 1:** **Number of participants involved in the study separated for Gender and different European Countries**.

**Country**	**Male**	**Female**	**N**
Austria	149	153	302
Bulgaria	386	57	443
Cyprus	56	47	103
Germany	217	96	313
Ireland	0	105	105
Italy	122	233	355
Latvia	108	64	172
Lithuania	222	241	463
Poland	198	227	425
N	1458	1223	2681

According to the reported personal driving experience, each participant was assigned to one of three different groups: Car drivers (participants who usually drive a car, even if they also occasionally ride a powered two wheeler—PTW), Motorcyclists (participants who usually ride a PTW but not a car), and Non-drivers (participants who drive neither a car nor a PTW). The motorcyclists were under-represented in the general sample compared the other groups. The study was performed according to the ethical principles expressed in the Declaration of Helsinki and it was approved by the local ethics committee (Psychology Department, University “Sapienza” of Rome, Italy). All participants provided their written consent to participate in the study and filled out a questionnaire with their socio-demographic information.

### Instruments and procedure

Participants were required to complete a questionnaire on basic demographic information and driving records, including an estimation of how many kilometers they drive weekly. The questionnaire was aimed at assessing attitudes toward road safety issues, driving behaviors in specific hypothetical situations, accident risk perception, and the concerns over these risks. The questionnaire contained the following measures:

#### Attitude toward road safety issues

(Driving Attitudes Scale—DAS—Iversen and Rundmo, 2004) This scale assesses road safety attitudes related to driving. Specifically, we assessed the attitudes toward rule violations and speeding, careless driving, as well as the attitude toward driving under the effects of alcohol and drugs (e.g., “Speed limits cannot be observed because they are too restrictive”). All the items were answered on six-point response scales ranging from “strongly disagree” (0) to “strongly agree” (5), with high scores indicating a negative attitude toward traffic safety (i.e., high preferences for risk-taking behavior).

#### Driver behaviour: violation, errors, and lapses (driver behaviour questionnaire–DBQ)

This scale is currently one of the most widely used scales for assessing self-reported driving behaviors (Lajunen and Summala, 2003). Respondents are required to indicate, on a six-point scale ranging from “never to” (0) to “nearly all the time” (5), how often they committed specific driving violations (12 items), errors (8 items), and lapses (8 items) in the past year.

#### Accident risk perception

Two items measuring risk perception were also included. On a ten-point response scale, ranging from “very low” (1) to “very high” (10), respondents were asked to evaluate their likelihood of being involved in a car accident compared to their fellows (i.e., “If you drive a car, how would you assess your risk of having a road accident, as compared to people of your age?”) and to indicate their level of concern about this possibility (i.e., “How much are you worried about this possibility?”).

The scales were almost identical for the three groups (Car drivers, Motorcyclists, and Non-drivers), with the exception that items were adapted for the specific group of respondents.

### Statistical analyses

Data from the different groups (Car drivers, Motorcyclists, and Non-drivers) were separately submitted to exploratory factor analyses, using the Principal Axis method and the oblique Oblimin rotation. This step was necessary, as not all the scales are validated for all the countries included in this study. The different scale factor scores were then computed through the regression method for each factor and used for further statistical analyses. Specifically, to examine possible differences between groups in driving attitudes and imagined driving behavior, factor scores from the two scales (Attitudes and Behaviors) were separately submitted to an Analysis of Variance (ANOVA) with Group (males, females) and Factors from each scale as independent variables. To examine possible differences between the groups in risk perception and the level of concern about this risk, the scores from the two Accident Risk Perception items (Personal Accident Probability and Level of Concern) were also submitted to a 2 (males, females) × 2 (Accident Probability and Concern) ANOVA.

To investigate differences in Attitude toward road safety for each Country, factor scores from the component of the Driving Attitudes Scale were submitted to a mixed-design 2 × 3 ANOVA with Gender as the independent variable and DAS components as dependent variables, for every individual Country. The sample of each Country was balanced for gender. In the case of Ireland it was not possible to proceed to the analysis, because men in the sample were not represented. We also investigated possible gender differences for each Country, in driving behavior, factor scores from the components derived from the factorial analysis of the Driving Behavior Questionnaire (DBQ) were submitted to a 2 × 2 ANOVA mixed design with Gender as the independent variable and DBQ Components as dependent variables for each Country.

## Results

### Factor analysis

#### Attitude toward road safety issues

Data from the Driving Attitudes Scale were submitted to exploratory factor analysis (Principal Axis method, Oblimin rotation). Measures of sampling adequacy, Kaiser-Meyer-Olkin = 0.854 and factorability of the correlation matrix, Bartlett's test of sphericity $\chi_{\left(153\right)}^{2}$ = 18350.62, *p* < 0.0001 were both adequate. The scree test yielded a third-factor solution accounting for 47.34% of the total variance. The first factor, labeled “Negative attitude toward traffic rules and risky driving,” accounts for 25.06% of the common variance and refers to the positive attitude toward risky driving behavior. Items such as “High-speed driving is possible if road conditions are good and there is nobody around” load on this factor.

The second factor, labeled “Negative attitude toward drugs and alcohol”, accounts for 15.37% of the common variance and refers to negative attitudes toward driving under the effects of psychoactive substances. Items such as “I would never drive after drinking alcoholic drinks” and “I would never drive under the influence of narcotic drugs” load on this factor. The third factor, labeled “Tolerance toward speeding”, accounts for 6.9% of the common variance and refers to a positive attitude toward riding in a car with a fast driver. Items such as “It is ok to ride in a car with a fast driver if it is the only way to go back home at night” load on this factor. This factor shows a slight positive correlation with the first factor (.42).

#### Driver behavior: violation and lapses

Data from the Driving Behavior Scale were submitted to exploratory factor analysis (Principal Axis method, Oblimin rotation). Measures of sampling adequacy, Kaiser-Meyer-Olkin = 0.973 and factorability of the correlation matrix, Bartlett's test of sphericity $\chi_{\left(561\right)}^{2}$ = 44853.656, *p* < 0.001 were both adequate. The scree test yielded a two-factor solution accounting for 48.49% of the total variance. The first, labeled “Errors in inattentive driving,” accounts for 40.38% of the common variance and refers to driving without respecting and paying attention to road rules. Items such as “Drive without keeping a safe distance” load on this factor. The second factor was labeled “Driving violations”. Items such as “You exceed the speed limit by 10 Km/h” load on this factor. This second factor positively correlates with the first factor (.6).

## ANOVA results

### Attitude toward road safety issues

**Figure 1:**
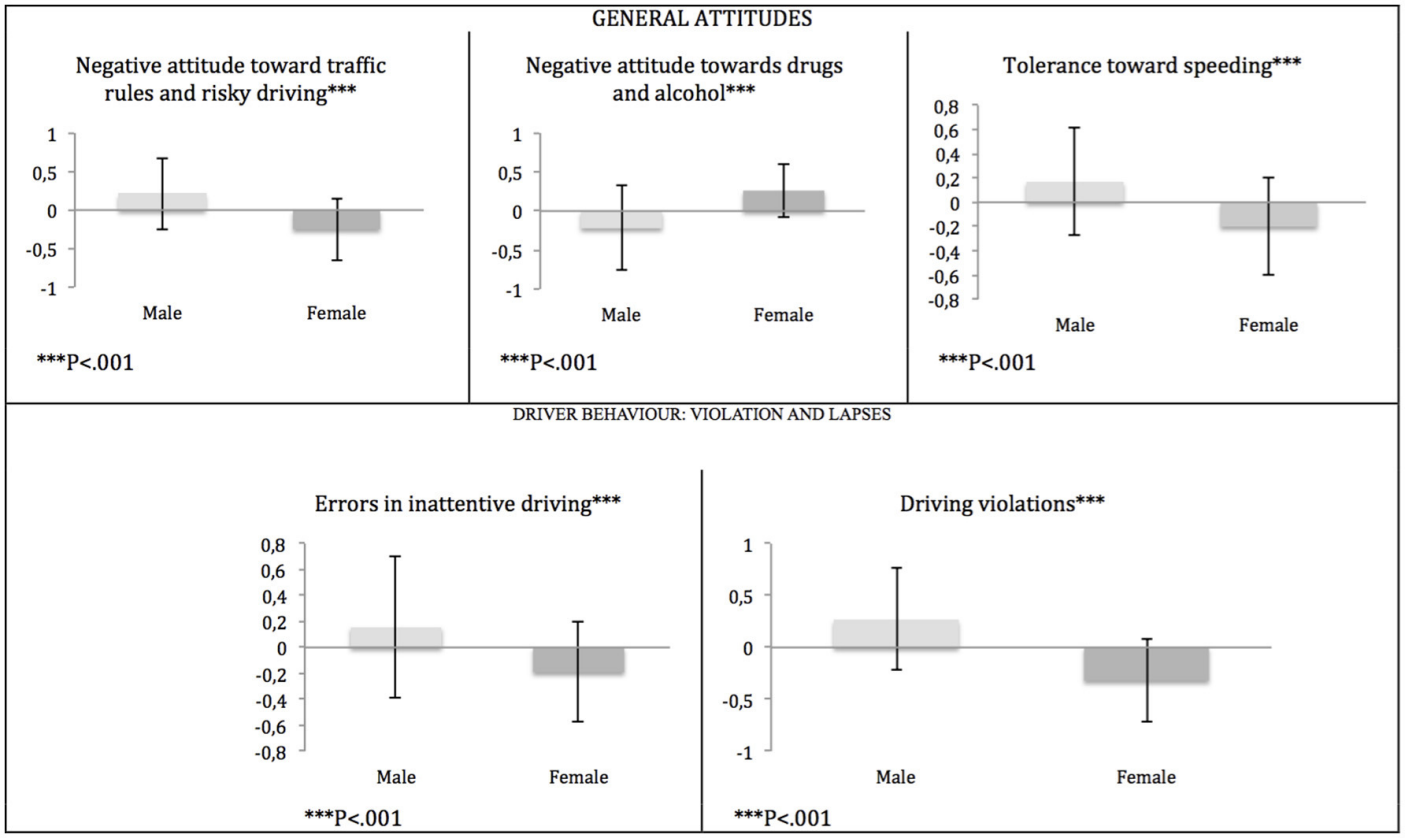
**Mean factor scores for the three dimensions derived from the General Attitudes on road safety, and for the two factors of the Driver Behavior Questionnaire**.

Factor scores from the component of the Driving Attitudes Scale were submitted to a mixed-design 2 × 3 ANOVA with Gender as the independent variable and DAS components (Negative attitude toward traffic rules and risky driving; Negative attitude toward drugs and alcohol; Tolerance toward speeding) as dependent variables. The ANOVA revealed a significant Gender X Component interaction, *F*_(2, 5212)_ = 225,389, *p* < 0.001 (Table 1 and Figure 1). Planned comparisons revealed that males had higher scores on the Negative attitude toward traffic rules component, *F*_(1, 2606)_ = 177.693, *p* < 0.001, *d* = −0.52, and for Tolerance toward speeding *F*_(1, 2606)_ = 125.210, *p* < 0.001, *d* = 0.53 (Table 2 and Figure 1), while in the Negative attitude toward drugs and alcohol component, females showed higher scores, *F*_(1, 2606)_ = 179.323, *p* < 0.001, *d* = −0.44]. These results show that male drivers are more prone to accept speeding, commit traffic violations, and use drugs and alcohol while driving.

**Table 2:** **Mean factor scores for the dimensions of the general attitudes and driver behavior are represented for gender**.

**GENERAL ATTITUDES**												
**Gender**	**Negative attitude toward traffic rules and risky driving**				**Negative attitude toward drugs and alcohol**				**Tolerance toward Speeding**			
	***n***	***M***	***(SD)***	**95% CI**	***n***	***M***	***(SD)***	**95% CI**	***n***	***M***	***(SD)***	**95% CI**
Male	1412	0.21	(0.92)	[0.16, 0.26]	1412	−0.22	(1.08)	[−0.27, −0.17]	1412	0.17	0.88	[0.13, 21]
Female	1196	−0.25	(0.81)	[−0.30, −0.20]	1196	0.26	(0.68)	[0.21, 31]	1196	−0.20	0.79	[−0.25, −0.15]
**DRIVER BEHAVIOR**												
**Gender**	**Errors in inattentive driving**				**Driving violations**							
	***n***	***M***	***(SD)***		**95% CI**	***n***	***M***	***(SD)***	**95% CI**			
Male	1367	0.16	1.09		[0.11, 0.21]	1367	0.27	(0.98)	[0.22, 0.32]			
Female	1149	−0.1, 91	0.76		[−0.25, −0.14]	1149	−0.32	(0.80)	[−0.37, −0.27]			

*Standard deviations (SD) and 95% Confidence Intervals are reported in brackets*.

### Driver behavior: violation and lapses

To investigate possible gender differences in driving behavior, factor scores from the components derived from the factorial analysis of the Driving Behavior Questionnaire were submitted to a 2 × 2 ANOVA mixed design with Gender as the independent variable and DBQ Components (Errors in inattentive driving, Driving violations) as dependent variables. The ANOVA revealed a significant Gender X Components interaction, *F*_(1, 2514)_ = 55.715, *p* < 0.001, indicating that the Gender shows different score trends in the two scale components. Planned comparisons revealed that male participants had higher scores in Errors in inattentive driving, *F*_(1, 2514)_ = 83.873, *p* < 0.001, *d* = −0.37, and in Driving violations, *F*_(1, 2514)_ = 262.603, *p* < 0.001, *d* = −0.65 (Table 2 and Figure 1).

Gender differences in attitudes and driving behavior for individual Country.

### Attitude toward road safety issues

#### Austria

In the case of Austria the ANOVA revealed a significant Gender X Component interaction, *F*_(2, 566)_ = 12.41, *p* < 0.001. Planned comparisons revealed that males had higher scores on the Negative attitude toward traffic rules component, *F*_(1, 283)_ = 17.402, *p* < 0.001, *d* = −5.94 (see Table 3), and Tolerance toward speeding *F*_(1, 283)_ = 4.34, *p* < 0.05, *d* = −0.25, while for Negative attitude toward drugs and alcohol females revealed higher scores *F*_(1, 283)_ = 8.202, *p* < 0.01, *d* = 0.34.

**Table 3:** **Contrast for gender and DAS components (Negative attitude toward traffic rules and risky driving; Negative attitude toward drugs and alcohol; Tolerance toward speeding) for each Country**.

**Country**	**Gender**	**Negative attitude toward traffic rules and risky driving**					**Negative attitude toward drugs and alcohol**					**Tolerance toward Speeding**				
		***n***	***M***	***SD***	***p***	**Cohen's *d***	***n***	***M***	***SD***	***p***	**Cohen's *d***	***n***	***M***	***SD***	***p***	**Cohen's *d***
**GENERAL ATTITUDES**																
Austria	Male	139	−0.005	0.087	<0.001	−5.94	139	−0.02	0.97	0.004	0.34	139	−0.07	0.89	0.038	−0.25
	Female	146	−0.47	0.069			146	0.26	0.64			146	−0.29	0.86		
Bulgaria	Male	59	−0.05	0.86	0.012	−0.48	59	−0.73	−1.15	0.009	0.50	59	0.06	0.79	0.364	0.17
	Female	55	−0.48	0.94			55	−0.14	−1.20			55	0.21	0.98		
Cyprus	Male	56	0.74	0.88	0.01	−0.51	56	−0.31	0.97	0.113	0.32	56	0.23	0.98	0.09	−0.33
	Female	47	0.25	1.02			47	0.003	0.99			47	−0.07	0.80		
Germany	Male	85	0.15	0.92	<0.001	−0.63	85	0.08	0.71	0.35	0.15	85	0.18	0.92	0.002	−0.47
	Female	93	−0.40	0.83			93	0.19	0.79			93	−0.22	0.78		
Italy	Male	121	0.16	0.93	<0.001	−0.7	121	−0.03	0.89	<0.001	0.49	121	−0.15	0.83	<0.001	0.92
	Female	118	−0.40	0.65			118	0.33	0.52			118	−0.52	0.60		
Latvia	Male	101	0.62	0.96	0.001	−0.56	101	−0.16	1.04	0.017	0.39	101	0.35	0.96	0.006	−0.45
	Female	63	0.11	0.80			63	0.21	0.81			63	−0.04	0.72		
Lithuania	Male	216	0.06	0.72	<0.001	−0.4	216	−0.21	1.10	<0.001	0.53	216	0.14	0.87	0.038	−0.19
	Female	237	−0.23	0.72			237	0.27	0.67			237	−0.02	0.84		
Poland	Male	197	0.57	0.88	<0.001	−0.7	197	0.003	0.95	<0.001	0.51	197	0.26	0.80	<0.001	−0.54
	Female	226	−0.02	0.80			226	0.37	0.44			226	−0.15	0.73		

#### Bulgaria

Also for Bulgaria the ANOVA showed a significant Gender X Component interaction, *F*_(2, 202)_ = 5.57, *p* < 0.01. Planned comparisons revealed a higher scores for the males on Negative attitude toward traffic rules component, *F*_(1, 112)_ = 6.52, *p* < 0.05, *d* = −0.48 (Table 3), while on the Negative attitude toward drugs and alcohol females showed higher scores *F*_(1, 112)_ = 7.072, *p* < 0.01, *d* = 0.34. There were not significant differences for the gender on factor Tolerance toward speeding.

#### Cyprus

The ANOVA showed a significant Gender X Component interaction, *F*_(2, 224)_ = 7.306, *p* < 0.01. Planned comparisons revealed that males had higher scores on the Negative attitude toward traffic rules component, *F*_(1, 101)_ = 6.772, *p* < 0.001, *d* = −5.94 (see Table 3), but no significant differences were found for the factors Negative attitude toward drugs and alcohol and Tolerance toward speeding.

#### Germany

Also for Germany we found significant differences in Gender X Component interaction, *F*_(2, 352)_ = 7.96, *p* < 0.001, and in the planned comparisons for the factors Negative attitude toward traffic rules component, *F*_(1, 176)_ = 17.39, *p* < 0.001, *d* = −0.63 (Table 3), and Tolerance toward speeding *F*_(1, 176)_ = 9.8, *p* < 0.01, *d* = −0.47. No significant differences were found for the Negative attitude toward drugs and alcohol.

#### Italy

Same result for the Italian sample: We found significant differences in Gender X Component interaction for the Italian sample, *F*_(2, 474)_ = 23.19, *p* < 0.001. Planned comparisons revealed that males had higher scores on the Negative attitude toward traffic rules component, *F*_(1, 237)_ = 29.28, *p* < 0.001, *d* = −0.7 (see Table 3), and Tolerance toward speeding *F*_(1, 237)_ = 15.972, *p* < 0.001, *d* = 0.92, while for Negative attitude toward drugs and alcohol females revealed higher scores *F*_(1, 237)_ = 14.54, *p* < 0.001. *d* = 0.49.

#### Latvia

The ANOVA presented a significant Gender X Component interaction, *F*_(2, 324)_ = 11.606, *p* < 0.001. Planned comparisons revealed that males had higher scores on the Negative attitude toward traffic rules component, *F*_(1, 162)_ = 12.347, *p* < 0.001, *d* = −0.56 (see Table 3), and Tolerance toward speeding *F*_(1, 162)_ = 7.8, *p* < 0.01, *d* = −0.45, while on the Negative attitude toward drugs and alcohol females showed higher scores *F*_(1, 162)_ = 5.79, *p* < 0.05, *d* = 0.39.

#### Lithuania

Also for Lithuania we found significant differences in Gender X Component interaction, *F*_(2, 902)_ = 28.84, *p* < 0.001. Planned comparisons showed that males had higher scores on the Negative attitude toward traffic rules component, *F*_(1, 451)_ = 18.96, *p* < 0.001, *d* = −0.4 (see Table 3), and Tolerance toward speeding F_(1, 451)_ = 4.32, *p* < 0.05, *d* = −0.19, while on the Negative attitude toward drugs and alcohol females showed higher scores *F*_(1, 451)_ = 32.6, *p* < 0.001, *d* = 0.53.

#### Poland

For the Polish sample we found a significant effect Gender X Component interaction, *F*_(2, 842)_ = 47.29, *p* < 0.001. Planned comparisons revealed that males had higher scores on the Negative attitude toward traffic rules component, *F*_(1, 421)_ = 51.73, *p* < 0.001, *d* = −0.7 (see Table 3), and Tolerance toward speeding *F*_(1, 421)_ = 30.03, *p* < 0.001, *d* = −0.54, while on the Negative attitude toward drugs and alcohol females showed higher scores *F*_(1, 421)_ = 27.67, *p* < 0.001, *d* = 0.51.

These results showed that for the negative attitude on traffic rules there are no differences for individual countries: Males drivers are generally more prone to no respect of the rules. Differently, for the Tolerance toward speeding and Negative attitude toward drugs and alcohol, we did not find gender difference in all countries.

### Driver behavior: violation and lapses

#### Austria

For the Austrian sample the ANOVA revealed a significant Gender X Component interaction, *F*_(1, 259)_ = 8.26, *p* < 0.01. Planned comparisons revealed that male participants had higher scores in Errors in inattentive driving, *F*_(1, 259)_ = 10.3, *p* < 0.001, *d* = −0.407, and in Driving violations, *F*_(1, 259)_ = 31.4, *p* < 0.001, *d* = −1.83 (Table 4).

**Table 4:** **Contrast for gender and DBQ Components (Errors in inattentive driving, Positive attitude toward traffic code, and Positive attitude toward Drugs and alcohol) for each Country**.

**DRIVER BEHAVIOR**											
**Country**	**Gender**	**Errors in inattentive driving**					**Driving violations**				
		***n***	***M***	***SD***	***p***	**Cohen's *d***	***n***	***M***	***SD***	***p***	**Cohen's *d***
Austria	Male	128	0.004	1.12	0.001	−0.407	128	0.21	1.04	<0.001	−1.83
	Female	133	−0.36	0.60			133	−0.41	0.70		
Bulgaria	Male	57	0.19	1.18	0.73	0.06	57	0.001	0.97	0.052	−0.37
	Female	56	0.27	1.24			56	−0.35	0.92		
Cyprus	Male	56	0.38	0.99	0.09	−0.33	56	1.25	0.83	<0.001	−0.82
	Female	46	0.05	0.98			46	0.50	0.99		
Germany	Male	86	−0.31	0.65	0.609	−0.08	86	−0.07	0.75	<0.001	−0.78
	Female	91	−0.36	0.51			91	−0.64	0.71		
Italy	Male	115	0.09	0.90	0.012	−0.30	115	0.12	0.90	<0.001	−0.69
	Female	111	−0.17	0.65			111	−0.49	0.64		
Latvia	Male	87	1	1.56	0.049	−0.34	87	0.85	1.22	0.001	−0.57
	Female	59	0.51	1.27			59	0.20	1.04		
Lithuania	Male	213	0.04	0.94	<0.001	−0.36	213	0.13	0.89	<0.001	−0.45
	Female	232	−0.25	0.64			232	−0.26	0.83		
Poland	Male	197	−0.32	0.62	0.33	−0.10	197	0.08	0.79	<0.001	−0.25
	Female	226	−0.38	0.56			226	−0.27	0.68		

#### Bulgaria

Also for Bulgaria the ANOVA showed a significant Gender X Component interaction, *F*_(1, 111)_ = 6.501, *p* < 0.05. Planned comparisons not revealed a significant differences in Errors in inattentive driving and in Driving violations (Table 4).

#### Cyprus

The ANOVA presented a significant Gender X Component interaction, *F*_(1, 100)_ = 6.46, *p* < 0.05. Planned comparisons revealed that males had higher scores in Driving violations, *F*_(1, 100)_ = 17.29, *p* < 0.001, *d* = −0.82 (Table 4). We have not found gender differences in the factor Errors in inattentive driving.

#### Germany

Also for Germany we found significant differences in Gender X Component interaction, *F*_(1, 175)_ = 33.09, *p* < 0.001, and in the planned comparisons for the factors Driving violations, *F*_(1, 175)_ = 27.02, *p* < 0.001, *d* = −0.78 (Table 4). We have not found differences in Errors in inattentive driving.

#### Italy

We found significant differences in Gender X Component interaction for the Italian sample, *F*_(1, 224)_ = 16.75, *p* < 0.001. Planned comparisons revealed that males had higher scores Errors in inattentive driving, *F*_(1, 224)_ = 6.34, *p* < 0.05, *d* = −0.3, and in Driving violations, *F*_(1, 224)_ = 33.9, *p* < 0.001, *d* = −0.69 (Table 4).

#### Latvia

The ANOVA showed a significant difference for the Gender X Component interaction, *F*_(1, 144)_ = 7.21, *p* < 0.01. Planned comparisons revealed that males had higher scores in Errors in inattentive driving, *F*_(1, 44)_ = 7.8, *p* < 0.01, *d* = −0.34, and in Driving violations, *F*_(144)_ = 3.95, *p* < 0.001, *d* = −0.57 (Table 4).

#### Lithuania

Also for Lithuania we found significant differences in Gender X Component interaction, *F*_(1, 443)_ = 25.87, *p* < 0.001. Planned comparisons showed that males had higher scores in Errors in inattentive driving, *F*_(1, 44)_ = 15.43, *p* < 0.001, *d* = −0.36, and in Driving violations, *F*_(144)_ = 23.78, *p* < 0.001, *d* = −0.45 (Table 4).

#### Poland

For the Polish sample we found a significant effect Gender X Component interaction, *F*_(1, 421)_ = 21.26, *p* < 0.001. Planned comparisons revealed that males had higher scores in Driving violations, *F*(1, 421) = 24.71, *p* < 0.001, *d* = −0.25 (Table 4). We have not found differences for the factor Errors in inattentive driving.

### Accident risk perception

**Figure 2:**
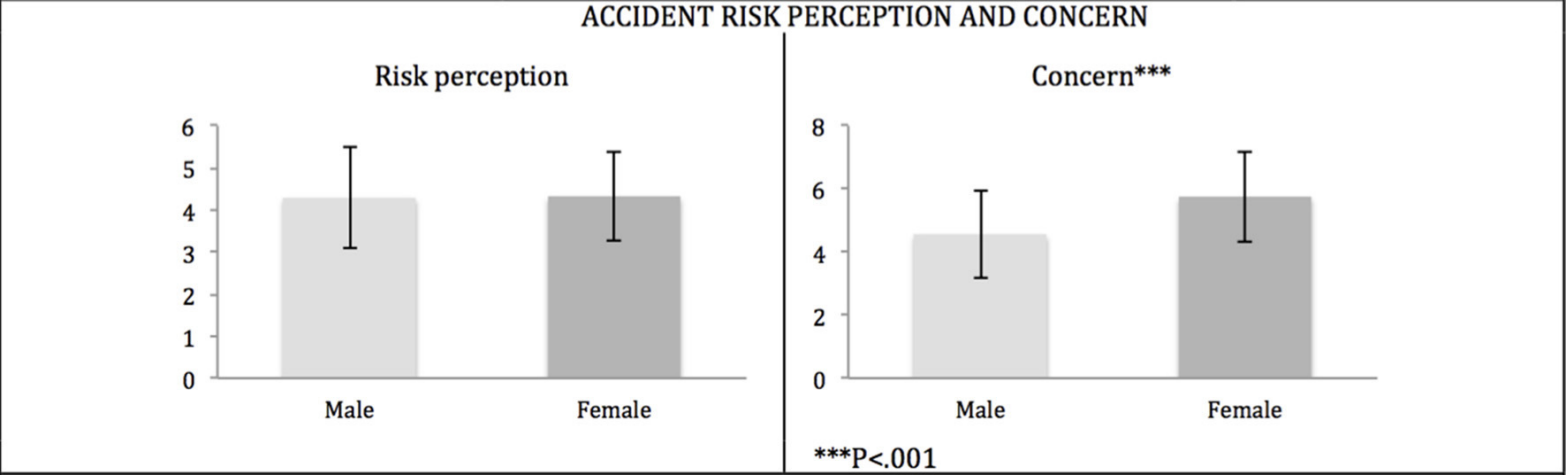
**Mean scores of the Accident Risk Perception and Risk Concerns are represented for each group of road users (Car drivers, Motorcyclists, and Non-drivers)**.

Scores from the two Accident Risk Perception items were submitted to a 2 × 2 mixed-design ANOVA, with Gender as the independent variable and Accident Risk (Probability and Concern) as dependent variables. The ANOVA yielded a significant main effect for Gender, *F*_(1, 2650)_ = 224.556, *p* < 0.001, and a significant Gender X Accident Risk interaction, *F*_(1, 2650)_ = 101.546, *p* < 0.001, revealing that gender shows different trends for the two risk measures. Contrasts revealed that, while females and males did not differ significantly in the level of accident risk perception, these two groups clearly show a significant difference regarding the level of concern about this risk, with males being significantly less concerned than females about the risk of a road accident, *F*_(1, 2652)_ = 115.552; *p* < 0.001, *d* = 0.42. This result is extremely interesting, as it shows that even if the accident risk perception is the same, males and females differ in their level of concern about this risk (see Figure 2).

## Discussion

According to Romano et al. (2008), the increase in the number of women involved in fatal crashes could be explained by an increase in traffic exposure but could also be related to the changes in the role of women in society, which in some way bring women to behave like men. However, these authors also observed that risky driver behaviors can be attributed only to young female drivers and not to older females overall.

For this reason, in the present study, we aimed to investigate the gender-related effects on road safety attitudes focusing on young drivers aged 18–22 years. Moreover, we performed this investigation in nine different European countries to observe also for the presence of social and cultural effects. The scientific literature suggests that young drivers are more likely to underestimate the risk of being involved in a crash and to overestimate their own abilities as drivers. Moreover, if it is true that women are masculinized in their driving behavior and that this phenomenon is more present in the youngest, we should not have found any type of gender effect in our sample.

Specifically, our results identify three main factors in the attitude toward road safety issues that characterize our sample. The first factor is “Negative attitude toward traffic rules and risky driving” in which participants justify unsafe behaviors in accordance with environmental situations. The second factor concerns “Negative attitude toward drugs and alcohol,” and the third factor is “Tolerance toward speeding.” With respect to Driver Behavior (i.e., violation and lapses), two main factors also emerged: “Errors in inattentive driving” and “Driving violations.”

Present results show that male young drivers are more prone to accept speeding, traffic violations and drugs and alcohol use by the driver. Moreover, concerning the negative attitude on traffic rules there are no differences among individual countries. Indeed, in all countries males are generally more prone to no respect the rules. Interestingly, for the Tolerance toward speeding and Negative attitude toward drugs and alcohol, we did not find gender difference in all countries. This finding may reflect differences in the use and consumption of alcohol and drugs among countries as well as in differences due to national policies adopted to reduce alcohol/drug-related harm due to their abuse.

In general, our finding is in line with the observation that females are less involved in alcohol-related crashes and speeding-related crashes than males (Kelley-Baker and Romano, 2010). Furthermore, Kelley-Baker and Romano (2010) found that many of these gender differences can be largely explained by gender differences in alcohol consumption. Our data seem to support this observation as well as the presence of gender effects regardless of changes in gender roles or in women's driving behavior.

However, in the current study, the most interesting results regarding gender differences concern the evidence that both men and women have the same perception regarding dangerous or risky situations, but only women showed concerns about perceived risk. Worry and perceived risks are often investigated together. Sjöberg (1998) distinguishes worry from risk perception in terms of emotion vs. cognition. Indeed, worry has been described in terms of emotional responses to a threat (e.g., affective responses), while perceived risk has been considered a cognitive assessment (e.g., perceptions of vulnerability). Our results showed that the main difference is in terms of emotions; indeed, women appear more worried than men with respect to risk perception as a cognitive evaluation.

It is known that perception of risk is multi-dimensional (Slovic et al., 1980) and includes factors like perceived personal controllability of the hazard and voluntariness of exposure to risky situations. Gender is another factor, as women rate hazards as riskier than do men (Slovic, 2000). However, what factors are related to risk perception, in what way and why have yet to be clarified (Hawkes and Rowe, 2008). Furthermore, future studies must take into account that risk perception changes over time (Hawkes et al., 2009). For this reason, we can hypothesize that younger drivers are very different from older drivers and that a study on risk perception that considers the aging effect will also be useful in developing targeted prevention programs. An interesting finding has come from research aimed to reduce medical errors. Peters et al. (2006) found that worry about medical errors was a better predictor of intention to take precautionary actions than risk perceptions. Furthermore, a positive association between worry and health-protective behavior has been observed (McCaul and Mullens, 2003). Several studies demonstrate that worry about breast cancer leads to precautionary behavior to prevent the illness (e.g., Wilcox et al., 2002; Cameron and Reeve, 2006). In a study aimed at investigating five domains of risk taking (i.e., financial decisions, health/safety, recreational, ethical and social decisions) found that women appeared to be more risk-averse in all domains except social risk (Weber et al., 2002).

Our findings seem to suggest that worry over risky situations may help in reducing hazardous behaviors. Both men and women were able to understand and to detect risk, but only women showed concern about the risk. Furthermore, as observed in our sample, men's driving behavior seems to confirm that young men are more prone to accept road violations and to justify alcohol consumption. Surely, prevention programs should consider these aspects and focus on worries that could increase careful driving behaviors.

## Author contributions

Conceived and designed the experiments: AMG, FF, RS, PC, FB, LP. Performed the experiments: PC, FB. Analyzed the data: PC. Wrote the paper: LP, PC, FB, AMG.

### Conflict of interest statement

The authors declare that the research was conducted in the absence of any commercial or financial relationships that could be construed as a potential conflict of interest.
